# Comparative Study on Two COVID-19 Outbreaks at a Long-Term Mental Health Facility in Korea in 2020 and 2022

**DOI:** 10.3390/medicina59061170

**Published:** 2023-06-18

**Authors:** Jina Kim, Gawon Choi, Jeonghyeon Oh, Kunhee Park, Seok-Ju Yoo

**Affiliations:** 1 Gyeonggi Infectious Disease Control Center, Health Bureau, Gyeonggi Provincial Government, Suwon 16508, Republic of Korea; tsjina0705@gmail.com (J.K.);; 2 Department of Preventive Medicine, College of Medicine, Dongguk University, Seoul 04620, Republic of Korea

**Keywords:** coronavirus disease 2019 (COVID-19), long-term, mental health facility, vaccination, outbreak

## Abstract

(1) *Background and Objectives***:** There were two distinct coronavirus disease 2019 (COVID-19) outbreaks in 2020 and 2022 at a long-term mental health facility (LTMHF) in Gyeonggi Province, Korea. We aimed to compare the two outbreaks and identify differences in epidemiological and clinical outcomes due to changes in epidemic timing and management methods. (2) *Materials and Methods*: The structural, operational, and case-specific LTMHF data of COVID-19-confirmed patients during these outbreaks in 2020 and 2022 were retrospectively analyzed. (3) *Results*: Forty individuals (37 residents) in 2020 and thirty-nine (32 residents) in 2022 were confirmed to have COVID-19, and ten were infected twice. Facility isolation was implemented as an infection control measure, and one COVID-19-related death occurred in 2020. All residents and staff were vaccinated at least twice in 2022; moreover, in 2022, 38 patients (97.4%) received a third vaccination less than months before infection. The average Ct value of the cases in 2022 was significantly higher than that in 2020; however, vaccine-breakthrough (V-BT) and reinfection after vaccination rates were similar. (4) *Conclusions*: COVID-19 vaccination could help lower the viral load of severe acute respiratory syndrome coronavirus 2 (SARS-CoV-2), which was inversely correlated with Ct values, and ventilation system improvements in health facilities might reduce transmissibility.

## 1. Introduction

During the coronavirus disease 2019 (COVID-19) pandemic, infections in long-term care facilities were an issue of considerable personal and social concern because of poor outcomes. Many studies have reported on the characteristics, preparedness, and prevention/mitigation of COVID-19 outbreaks in long-term care facilities for the elderly [1]; however, relatively few have studied long-term mental health facilities (LTMHFs) [2,3]. Unlike long-term elderly care facilities, LTMHFs accommodate patients with mental illness in closed wards, and patients are cared for by mental health-related experts such as psychiatrists, nurses, nurses’ aides, social workers, mental health specialists, and mental health instructors [4].

Two years after the onset of the COVID-19 pandemic in Korea in early 2020, the number of infected people increased significantly; a total of 139,466 cases and 112 deaths were recorded by 1 March 2022 [5], despite the implementation of countermeasures, such as social distancing, personal hygiene, and extensive national vaccination campaigns. This explosive increase is presumed to have been due to the waning of antibody titer to the virus, the emergence of variants of concern (VOCs), fatigue-induced noncompliance with social measures, and a substantial increase in the number of vaccine-breakthrough (V-BT) infections [6].

In January 2022, 16 months after the first outbreak in September 2020, the second COVID-19 outbreak occurred in the same LTMHF in Gyeonggi-do, South Korea. Although there was no significant change in the organization’s structure, organization, residents, or number of employees during the 16 months, all participants in this study received at least two COVID-19 vaccines under the Korean health authorities’ quarantine policy for vulnerable facilities. Most of the residents in the LTMHF suffer from schizophrenia, and those suffering from schizophrenia in the general public are 4.09 times more likely to die when infected with COVID-19 [7].

This first study of the COVID-19 outbreaks in an LTMHF in Korea aimed to analyze epidemiological and clinical characteristics such as the severity of illness reported in COVID-19-positive people using PCR tests conducted during the primary and secondary pandemics and Ct values of target genes and to compare the results. It also proposes appropriate measures for infection control.

## 2. Materials and Methods

### 2.1. Data Collection

We retrieved data on confirmed cases from the COVID-19 reporting and surveillance system operated by the Korea Disease Control and Prevention Agency and the Health and Medical Crisis Response System by the Health Insurance and Review Assessment (HIRA) Service [8,9]. Age, sex, symptoms, date of symptom onset, specimen type, date of specimen collection, date of diagnosis, Ct values for RdRp and E target genes, underlying disease, and vaccination history were obtained and subjected to demographic and clinical analysis. The facility manager provided information on the structure and operation of the LTMHF and on patient and contact management. In addition, information on structural arrangements, numbers of rooms and beds, numbers and locations of bathrooms, work schedules, places supervised by staff members, and daily surveillance reports during facility isolation were reviewed. Viral sequencing analysis was not performed during either of the two outbreaks.

### 2.2. Statistical Analysis

For patient demographic and clinical findings, categorical variables are presented as numbers and proportions, and continuous variables are presented as averages and standard deviations. To compare Ct values, we classified confirmed cases into three groups according to vaccination history, i.e., unvaccination in 2020, first infection after vaccination(V-BT) in 2022, reinfection after vaccination(V-BT/R) in 2022; V-BT, infected once in 2022 and V-BT/R, infected each once in 2020 and 2022. The analysis was conducted using Student’s t-test, one-way analysis of variance, and Mann–Whitney U test. Data were processed using Microsoft Excel (Microsoft Corp., Redmond, WA, USA) and R version 4.0.3 (R Foundation for Statistical Computing, Vienna, Austria), IBM SPSS Statistics version 25.0 and statistical significance was accepted for *p* values < 0.05.

### 2.3. Ethics Approval

This study was approved by the Bioethics Committee of public institutions designated by the Ministry of Health and Welfare to exempt written consent under Paragraph 3 of Article 16 of the Bioethics and Safety Act (approval number: P01-202203-01-036). First, the minimum age of the subjects of this study was 29 years old (workers), and children were not included. Second, it falls under both 1 and 2 of Paragraph 3 of Article 16 of the Bioethics Act (Consent of Human Research).

## 3. Results

### 3.1. Outbreak Settings

**Figure 1:**
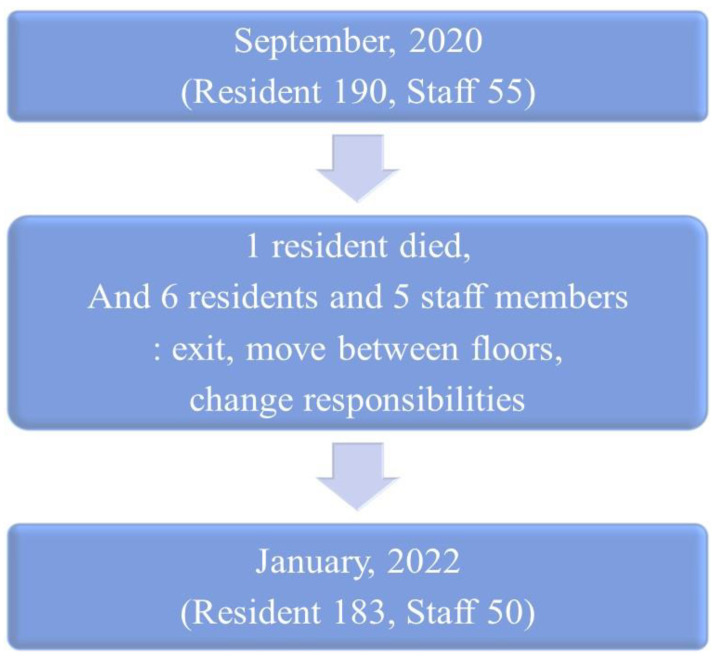
During the first epidemic process in September 2020, changes in members of the facility occurred, showing a difference from the number of people subject to quarantine during the second epidemic in January 2022.

**Figure 2:**
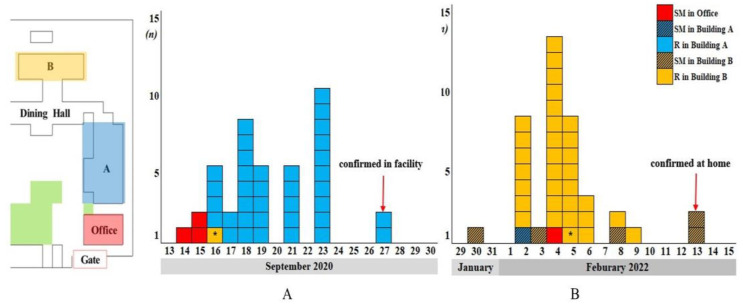
Location of outbreaks and epidemic curves in 2020 and 2022. The first COVID-19 outbreak occurred in building A, third floor, and 36 residents (62.07%) were infected (**A**). The second outbreak occurred in building B, second floor, and 31 (77.50%) residents were infected. Moreover, all 39 were vaccine-breakthrough (V-BT) infections (**B**). Times between confirmations of infections in first and last patients were 13 days in 2020 and 11 days in 2022. SM, staff member; R, resident; *, female.

The LTMHF comprises three buildings, namely, buildings A, B, and an office, capable of accommodating 300 residents and approximately 60 staff members (Table 1 and Figure 1 and Figure 2). Males and females use separate floors in each building. Building A is a three-story building with 10 rooms on the second and third floors. There are no beds or bathrooms in the rooms; thus, all residents use a communal area on each floor. Building B is a two-story building with a structure similar to that of building A and is operated in the same manner; however, the latter has seven rooms per floor. Buildings A and B are connected to a large dining hall by stairs and hallways. Residents were sometimes moved to different rooms and buildings according to medical situations (Figure 2). The isolation subjects included 190 residents and 55 staff during the first epidemic in October 2020; however, there were 183 residents and 50 staff during the second pandemic in January 2022 due to the death of one of the residents, leaving and moving between floors, and the changing of duties (Figure 1).

### 3.2. Epidemiologic and Clinical Findings of COVID-19 Outbreaks in 2020 and 2022

The first outbreak occurred on 14 September 2020, when a 57-year-old facility director received a confirmed diagnosis of COVID-19 based on a positive severe acute respiratory syndrome coronavirus 2 (SARS-CoV-2) polymerase chain reaction (PCR) test three days after symptom commencement. Screening conducted the following day identified positive for COVID-19 of staff members who used the same office as the director, as well as, residents in buildings A and B (Figure 2A). Facility and home isolation were implemented as infection control measures and were discontinued 14 days after the last infected person in the facility had a confirmed diagnosis. In 2020, a total of 40 individuals with an average age of 55.96 (±9.52) years contracted COVID-19: 37 (92.5%) were residents, and 3 (7.5%) were staff members. Of the residents, 36 (90.0%) were men living on the third floor of building A and 1 was a woman living on the first floor of building B. Twenty-four (60%) of these forty individuals were asymptomatic at diagnosis, and thirty-nine (97.5%) had a mild disease during hospitalization. However, one resident (2.5%) died of SARS-CoV-2-related pneumonia. Regarding underlying diseases, three patients (7.5%) had hypertension, four patients (10.0%) had diabetes mellitus, and five patients (12.5%) had hyperlipidemia. All residents had mental problems: 32 had schizophrenia (80.0%), and 5 had unspecified hallucinations, delusions, panic disorder, or paranoia. No vaccination history was recorded for the 2020 outbreak (Table 2).

The second outbreak started when a 39-year-old social worker in charge of the residents on the second floor of building B (Figure 2B) received a positive SARS-CoV-2 PCR test result on 30 January 2022. He complained of a fever that had occurred 2 days previously. Subsequently, additional confirmed cases were identified via PCR screening. 

Facility and home isolation were implemented to block further spread within the facility. Countermeasures were terminated 7 days after the last positive PCR test at the facility. Thirty-two (82.1%) of the thirty-nine confirmed patients were residents, and thirty-five (89.7%) were men. Seventeen patients (43.6%) had symptoms at the time of infection confirmation. All patients were asymptomatic or had a mild hospital course. Including redundancy, in 39 patients of this cluster, 6 (15.4%) had hypertension, 3 (7.7%) diabetes mellitus, and 14 (35.9%) had hyperlipidemia. All residents had mental problems or schizophrenia. All 39 patients received two or more doses of the vaccine, and 38 (97.4%) had a third vaccination history (the injection date of the third dose was November 2021). One patient that had been vaccinated twice was a new social worker who received a second vaccination on 16 January 2022 (Table 2).

Of the 39 confirmed patients, 10 were infected both in 2020 and 2022. All patients were male residents with an average age of 56.8 (±4.24) years.

### 3.3. Comparison of Viral Ct Values in 2020 and 2022

**Figure 3:**
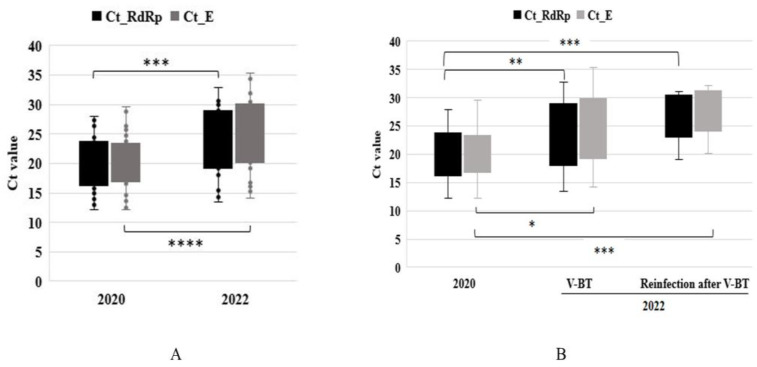
Boxplot graph of Ct values for the RdRp and E genes in 2020 and 2022. Mean Ct values in 2020 (*n* = 40) and 2022 (*n* = 39) were significantly different (**A**), as were those in the 40 cases in 2020 (*n* = 40) and in the V-BT group in 2022 (*n* = 29), as well as in the 40 cases in 2020 and in the reinfection group in 2022 (*n* = 10) (**B**). Levels of significance are indicated as follows: ns, *p* > 0.05; *, *p* ≤ 0.05; **, *p* ≤ 0.01; ***, *p* ≤ 0.001; ****, *p* ≤ 0.0001.

The mean Ct values in 2020 for the 40 patients that were unvaccinated were 20.13 (±4.18) for the RdRp gene and 20.05 (±4.69) for the E gene, and these values were significantly different from those of the 39 fully vaccinated patients in 2022 (23.95 (±5.42) and 25.01 (±5.54), respectively) (Figure 3A). In 2022, the Ct values of the 29 unvaccinated patients in the V-BT group (mean Ct value: RdRp 23.30 (±5.74), E gene group 24.38 (±5.93)) the and the 10 patients in the V-BT reinfection group (mean Ct value: RdRp 26.05 (±4.02), E gene 27.15 (±3.91)) were also significantly different (Figure 3B). However, in 2022, the Ct values for different numbers of infections were not statistically significant (Figure 3B). The Ct values of 10 residents reinfected in 2020 and 2022 (mean Ct value in 2020: RdRp 22.38 (±3.89), E gene 22.87 (±3.75); in 2022: RdRp 26.05 (±4.23), E gene 27.15 (±4.12)) were non-significantly different, as determined using a Mann–Whitney U test (*p* = 0.08 for the RdRp gene and 0.03 for the E gene).

## 4. Discussion

In this study, we analyzed the data of individuals that contracted COVID-19 during outbreaks 16 months apart at an LTMHF in Gyeonggi Province, Korea. In these two outbreaks, which started from staff members, the total numbers of infected persons in 2020 and 2022 were 40 and 39, respectively. Infection rates were similar at 16.33% and 16.74%, respectively, despite an intervening public health authorities’ vaccination campaign. However, the mean Ct values of patients, including V- and reinfection after vaccination cases, were significantly different in 2020 and 2022. We believe that the high Ct value obtained in 2022 was due to the effect of vaccination because there had been no changes in staff or patient numbers in the facility. 

Real-time reverse transcription-PCR (RT-PCR) is a standard diagnostic test for SARS-CoV-2 infection. However, comparative analyses of Ct values must be performed carefully due to their semi-quantitative nature. In addition, diagnostic testing using commercially available kits produces heterogeneous results through suboptimal inter-test agreements. Therefore, Ct values are recommended for patient management [10,11]. Many studies have been conducted on the relationships between Ct values and the infectivity, disease severity, and fatality rate of SARS-CoV-2 infections by assuming an inverse linear correlation between Ct values and viral load [12]. Moreover, studies which have used Ct values have concluded that vaccines are effective. Several studies conducted in the United Kingdom and the United States have reported an increased risk of death in patients with high viral load, as indicated by a lower cycle threshold (Ct value) in RT-PCR tests [13,14].

The results of Atul K et al.’s cohort study during the third wave in Western India also showed an increase in mortality in patients with low Ct values through univariate analysis and adjusted logistic regression, supporting the idea that the vaccination protects against severe illness, hospitalization, and death caused by COVID-19 [15]. Furthermore, the Ct values of vaccinated individuals tend to be higher than those of unvaccinated individuals [16,17] and increase more rapidly after symptom onset, which supports the notion that viral load decreases more rapidly in vaccinated patients [18].

In Korea, if a rapid antigen test conducted by a medical expert since March 2022 is confirmed to be positive, the patient is recognized as a COVID-19-confirmed patient [19]. This is to respond early to infectious diseases by applying RAT, which can be diagnosed relatively faster than PCR, even if false positives are considered as the COVID-19 omicron variant spreads rapidly.

When the institution experienced the COVID-19 epidemic, there was no time to apply RAT to conduct diagnoses; moreover, considering that it was difficult for residents to accurately express their physical condition as disabled people, it was necessary to predict or manage the patients’ symptoms based on the Ct value of the PCR test. However, if RAT could have been used for diagnosis at this time, infection control methods such as immediate isolation and the additional management of contacts could have been applied based on rapid test results, epidemiological relevance, and clinical symptoms.

Emerging SARS-CoV-2 variants had a major impact on the COVID-19 pandemic, which has now persisted for over 2 years [20]. Ct values of variants continue to be used in studies on viral dynamics and comparative studies on variant types [21,22,23]. Reported Ct values of alpha variant-infected cases are lower than those of delta variant-infected cases [20,21]; moreover, they are significantly lower for cases infected with the omicron variant, which was the dominant strain in early 2022, compared to alpha variant-infected cases [23]. Selective viral sequencing for SARS-CoV-2-CoV-2 showed that the wild type accounted for 100% of cases in September 2020 and that the omicron variant accounted for 86.32% of cases on the third week in January 2022 in Gyeonggi Province [9]. Although no variant testing was performed in our cases, it is likely that the wild type and omicron variants were responsible for the LTMHF outbreaks in 2020 and 2022, respectively. Given that available results of comparative analyses on the Ct values of individuals infected with the wild type or omicron variant have not been determined, we considered our results intriguing. Despite the limitations associated with PCR testing at different laboratories in 2020 and 2022, based on the likelihood that the viruses responsible differed, and that all residents and staff members completed their vaccination courses in 2022, our results suggest that vaccination would have influenced Ct values and lowered viral load during infection.

Two reports published in Korea during the outbreak of COVID-19 in psychiatric hospitals relate to cases of COVID-19 in two different closed psychiatric wards before vaccination in February 2020 and after vaccination in October 2021 [24,25]. Reported fatality rates were 6.9% and 1.2%, respectively. By analyzing cluster infections in October 2021, Wi et al. concluded that, while vaccination could reduce mortality and the duration of hospitalization, it could not prevent SARS-CoV-2 delta variant outbreaks in contemporary psychiatric hospital settings [24]. In the current study, all COVID-19-confirmed patients in 2022 had received a vaccination less than three months before infection, and fatality rates were 2.5% in 2020 and 0% in 2022.

Infection control measures against COVID-19 outbreaks in elderly long-term care facilities have been meticulously studied [26,27,28]. A high baseline level of alertness involving visitor limitations, regular education, training to deal with COVID-19, restricted access to the outside world, and adaptive management strategies are implemented to contain outbreaks after the detection of a first infector are mandatory for infectious disease control in this environment [26,27,28]. Countermeasures for infection control in mental care facilities are similar to those implemented in nursing facilities for the elderly. Xiong et al. suggested that universal testing is likely to be the most reliable method of detecting SARS-CoV-2 because of the limited assistance of symptom reporting and medical monitoring in a community of an inpatient long-term care psychiatric rehabilitation facility [3]. Nava et al. reported that systemic guidelines at a psychiatric long-term care rehabilitation facility effectively prevented infection [2]. Both outbreaks analyzed in the present study were initiated by staff members, i.e., a director in 2020 and a social worker in 2022. In the first outbreak, the spread of infection from the facility director was presumed to have progressed to male and female residents via nurses working in the same office (Figure 2A). The second outbreak was initiated by a male social worker in building B and spread to male and female residents via social workers on the same floor and in another building (Figure 2B). Because those affected were in close daily contact, respiratory droplets and direct contact were the probable transmission routes; however, rather surprisingly, no additional spread occurred among female residents on the first floor of building B. These observations emphasize the importance of a higher baseline level of alertness for staff members than residents. 

The transmission of SARS-CoV-2 by respiratory droplets and direct close contact has been well established, as has viral transmission in aerosols, which increases the risk of spread in poorly ventilated spaces [29]. Despite the importance of ventilation, facilities at the studied LTMHF were worrisome. An epidemiological investigation revealed the absence of central air purification systems and a dependence on doors and windows for ventilation. However, doors were almost always closed and strictly controlled by facility managers; moreover, in one LTMHF, there was only one window measuring 0.8 m × 0.25 m in a 7.2 m × 4.5 m-sized room that housed eight residents. Window sizes are deliberately limited to prevent resident deviance and accidents. Chun et al. highlighted the problems posed by sealed windows and poor ventilation during the COVID-19 outbreak in a psychiatric hospital [25]. In addition, it has been reported that, during omicron variant outbreaks, fully vaccinated individuals can release virus-containing aerosols during breathing even 2 weeks after symptom onset [30]. Therefore, upon thorough onsite investigation of situations with an elevated risk of airborne transmission, we emphasize the need for ventilation system improvements.

## 5. Conclusions

The incidence of COVID-19 is increasing volatile, and viral variants continue to evolve. As a result, outbreaks in long-term care facilities have become a major social issue. We describe two COVID-19 outbreaks in a special facility for mentally disabled patients and summarize epidemiological characteristics.

Although only one facility is evaluated in detail and the number of subjects is limited, the results of this study support the effect of vaccination. It is also significant in that the study was conducted with similar subjects in the same long-term mental health facility during the historical epidemic of COVID-19. In addition, reports on outbreaks in mental care facilities are scarce; thus, we believe this study aids in the recognition and resolution of structural problems.

In addition to COVID-19, we suggest additional studies to confirm vaccination history and target gene levels of respiratory infectious diseases such as chickenpox and measles.

## Figures and Tables

**Table 1:** COVID-19 outbreak setting in an LTMHF and outbreak results in 2020 and 2022.

		September 2020	January 2022
**Outbreak settings**			
Persons (*n*)	Residents	190	183
	Building A	Second floor: 59 Third floor: 58	Second floor: 54 Third floor: 51
	Building B	First floor: 35 Second floor: 38	First floor: 38 Second floor: 40
	Staff members	55	50
	Total	245	233
Vaccination history (%)	Residents	No (0)	Yes (100) Complete a third dose ^1^
	Staff members	No (0)	Yes (100) Complete a third dose (96) ^1^ Complete a second dose (4) ^2^
**Outbreak results**			
Index characteristics		A 57-year-old director, Female	A 39-year-old social worker, Male
Facility implementations		Cohorting of residents and home isolation of staff members	
Attack Rate (%)	Residents		
	Building A	third floor: 62.07 (36/58)	third floor: 0
	Building B	first floor: 2.80 (1/35)	first floor: 2.63 (1/38)
		second floor: 0	second floor: 77.50 (31/40)
	Staff members	5.45 (3/55)	14.00 (7/50)

^1^ Adenovirus vector/adenovirus vector/mRNA. ^2^ Adenovirus vector/Adenovirus vector or mRNA/mRNA.

**Table 2:** Demographic and clinical findings of COVID-19-confirmed patients in the 2020 and 2022 outbreaks.

		September 2020 (*n* = 40)	January 2022 (*n* = 39)	*p*-Value
Age, yrs (mean ± SD)		55.98 (±9.52)	54.46 (±10.94)	0.3903
	Residents	57.03 (±8.53) (*n* = 37)	56.08 (±9.69) (*n* = 32)	0.8292
	Staff members	57.71 (±11.43) (*n* = 3)	54.46 (±11.09) (*n* = 7)	0.4751
Sex(*n*/%)				1.0000
	Male	37/92.5	35/89.7	
	Female	3/7.5	4/10.3	
Role				0.29
	Residents	37	32	
	Staff members	3	7	
Vaccination status (*n*/%)				
Completion of three doses		0	38/97.4 ^1^	
Completion of two doses		0	1/2.6 ^2^	
Symptoms at diagnosis (*n*/%)				0.9241
Yes		16/40	17/43.6	
No		24/60	22/56.4	
In-hospital course (*n*/%)				
Asymptomatic/Mild		39/97.5	39/100	
Severe(days confirmation to death)		1 died (8 days)/2.5	0	
Underlying disease (*n*/%)				
Hypertension		3/7.5	6/15.4	
Diabetes mellitus		4/10.0	3/7.7	
Hyperlipidemia		5/12.5	14/35.9	
Mental disorder		37	32	
-Schizophrenia		32	32	
-Non-schizophrenia		5 ^3^	0	
Ct value				
Ct_RdRp, mean (±SD)		20.13 (±4.18)	23.95 (±5.42)	
Ct_E, mean (±SD)		20.05 (±4.69)	25.01 (±5.54)	

^1^ Adenovirus vector/adenovirus vector/mRNA. ^2^ mRNA/mRNA. ^3^ Unspecified delusion and hallucination, paranoid, and panic disorder.

## Data Availability

The data used in this study are protected under the Personal Information Protection Act.
